# Intestinal strongyloidiasis and hyperinfection syndrome

**DOI:** 10.1186/1476-7961-4-8

**Published:** 2006-05-30

**Authors:** Raja S Vadlamudi, David S Chi, Guha Krishnaswamy

**Affiliations:** 1 Department of Internal Medicine, Quillen College of Medicine, East Tennessee State University, VA Building 1, Johnson City, Tennessee, USA; 2 Division of Allergy and Immunology, James H. Quillen Veterans Affairs Medical Center, Johnson City, Tennessee, USA

## Abstract

In spite of recent advances with experiments on animal models, strongyloidiasis, an infection caused by the nematode parasite *Strongyloides stercoralis*, has still been an elusive disease. Though endemic in some developing countries, strongyloidiasis still poses a threat to the developed world. Due to the peculiar but characteristic features of autoinfection, hyperinfection syndrome involving only pulmonary and gastrointestinal systems, and disseminated infection with involvement of other organs, strongyloidiasis needs special attention by the physician, especially one serving patients in areas endemic for strongyloidiasis. Strongyloidiasis can occur without any symptoms, or as a potentially fatal hyperinfection or disseminated infection. Th_2 _cell-mediated immunity, humoral immunity and mucosal immunity have been shown to have protective effects against this parasitic infection especially in animal models. Any factors that suppress these mechanisms (such as intercurrent immune suppression or glucocorticoid therapy) could potentially trigger hyperinfection or disseminated infection which could be fatal. Even with the recent advances in laboratory tests, strongyloidiasis is still difficult to diagnose. But once diagnosed, the disease can be treated effectively with antihelminthic drugs like Ivermectin. This review article summarizes a case of strongyloidiasis and various aspects of strongyloidiasis, with emphasis on epidemiology, life cycle of *Strongyloides stercoralis*, clinical manifestations of the disease, corticosteroids and strongyloidiasis, diagnostic aspects of the disease, various host defense pathways against strongyloidiasis, and available treatment options.

## Background

In United States, strongyloidiasis is the most important nematode infection in humans with a tendency towards chronic persistent infection and with special characteristic features of autoinfection, hyperinfection involving pulmonary and gastrointestinal systems, and disseminated infection involving other organs [1-4]. Strongyloidiasis is caused by a soil dwelling nematode helminth, *Strongyloides stercoralis*. This helminth resides in the small intestine of the human host. There is another species of same genus, *Strongyloides fuelleborni *that can also cause human infection but is mostly seen in African countries [4].

Infection with *Strongyloides stercoralis *was first reported in the year 1876 in French soldiers working in Vietnam [4]. It took nearly 50 years for the complete elucidation of the complex life cycle after the discovery of the parasite [4] because of the rare and characteristic feature of autoinfection that occurs in the life cycle. Strongyloidiasis was first described by Fulleborn in 1926 [5]. First reports of disseminated infection or hyperinfection date back to 1966 when Cruz et al., and Rogers et al., independently documented the occurrence of fatal strongyloidiasis with immunosuppression [6,7].

Though many advances have been made in the diagnosis and treatment of strongyloidiasis, it still prevails as one of the elusive diseases to tackle in the present day world. Strongyloidiasis may have a spectrum of manifestations ranging from the most common asymptomatic disease to potentially life threatening hyperinfection syndrome and disseminated disease. The patients, if symptomatic, present with pulmonary and gastrointestinal symptoms. Most of them are found to have strongyloidiasis after a laboratory work up reveals an incidental finding of eosinophilia. This review article documents a case report with symptoms along with review of the epidemiology, biology of strongyloidiasis, clinical manifestations of the disease including hyperinfection syndrome, effect of systemic corticosteroids on strongyloidiasis, diagnostic aspects of the disease, various pathophysiological mechanisms and host defense pathways regulating strongyloidiasis, and different options available to treat the infection.

## Case report

A 77 year old male veteran with past medical history significant for chronic obstructive pulmonary disease, coronary artery disease status post coronary artery bypass graft, dyslipidemia, hypertension, and gastro esophageal reflux disease was found to have an incidental eosinophilia with 12.4% eosinophils (absolute eosinophil count of 800 cells/mm^3^). He reported morning cough with small amounts of thick mucus. He denied any epistaxis, difficulty in breathing, abdominal pain, diarrhea and constipation. He had lived in North East Tennessee for almost 30 yrs. He was an ex-smoker but had ceased to smoke almost 30 years ago. On examination, he was an obese male with periorbital edema. Auscultation demonstrated a bruit just above the left sternoclavicular joint, but his chest was clear to auscultation. A midline scar consistent with previous coronary bypass grafting was seen. The rest of the examination was essentially benign.

Due to eosinophilia, a complete evaluation was carried out. Serological tests for strongyloidiasis were strongly positive with antibody titer of 12.2 (Normal titer < 1.0). Total serum levels of IgE and IgA were within-normal limits at 130 IU/mL and 243 mg/dL respectively and no *Strongyloides stercoralis *larvae or eggs were found in the stools as shown in the Table 1. He was given one dose of ivermectin (200 micrograms/kilogram) and the repeat labs, a month later, showed improved eosinophil percentage at 5.6% with a drop in the eosinophil count to 400 cells/mm^3 ^along with drop in the strongyloid antibody titer to 6.76 (Normal titer < 1.0) as shown in the Table 1. Three months after treatment, his respiratory symptoms improved and one more dose of ivermectin (200 micrograms/Kg) was given for still elevated strongyloid antibody titer of 6.97 (normal titer < 1.0). Repeat labs, 4 months after the second treatment with ivermectin, showed decreased strongyloid antibody titer to 5.0 (normal titer < 1.0) with no eosinophilia and thus indicating a positive response to treatment (Table 1).

**Table 1:** Laboratory Findings of Case Report

**Lab Finding**	**Before Rx**	**One Month After 1^st^Rx**	**Four Months After 2^nd^Rx^§^**
Sodium (mEq/L)	146	142	140
Potassium (mEq/L)	4.5	5.3	4.3
Chloride (mEq/L)	105	102	106
Carbon Dioxide (mEq/L)	28	29	27
Glucose (mg/dL)	93	107	93
Blood Urea Nitrogen (mg/dL)	18	20	17
Creatinine (mg/dL)	1.1	1.3	1.3
Calcium (mg/dL)	9.1	9.3	8.9
Albumin (g/dL)	4.1	3.9	*
Total Protein (g/dL)	7.8	7.5	*
Alkaline Phosphatase (U/L)	74	75	*
SGPT (U/L)	18	14	*
SGOT (U/L)	18	21	*
Eosinophils (%)	**12.4**	**5.9**	**5.2**
Absolute Eosinophil Count (cells/mm^3^)	**800**	**400**	**300**
Strongyloid Antibody by ELISA (Index)	**12.20**	**6.76**	**5.0**
IgE (IU/mL)	130	*	*
IgA (mg/dL)	243	*	*
*S. stercoralis *Larvae/Eggs in Sputum and Stools	Negative	Negative	*

*, Not available; Rx, Treatment with Ivermectin; SGPT, Serum Glutamic-Pyruvic Transaminase; SGOT, Serum Glutamine-Oxaloacetic Transaminase;

IgE, Immunoglobulin E; IgA, Immunoglobulin A; ^§^, Second treatment with ivermectin is given 3 months after the first treatment;

This is a case of mildly symptomatic chronic strongyloidiasis with very few respiratory symptoms consisting of morning cough with small amounts of thick sputum, positive strongyloid antibody in the serum and negative larvae or eggs in the stool samples. This case also demonstrates the effectiveness of ivermectin in the treatment of strongyloidiasis.

## Epidemiology

Though parasitic infections are rare in the developed world, sporadic cases of strongyloidiasis, toxocariasis, and giardiasis happen especially in the endemic areas. Physicians should be aware of the endemic areas for strongyloidiasis because of difficulty in diagnosis and high potential for fatal complications [2]. The parasite is mostly confined to the tropics and subtropics infecting about 100 million people in about 70 countries [4]. It is endemic in Southern, Eastern, and Central Europe, Islands of the Caribbean, Latin America, Sub-Saharan Africa and Southeast Asia [1,4]. In non-endemic regions of the world, it is diagnosed in the prisoners of war (POWs) of World War II and immigrants from endemic countries [8]. In the United States, the infection is more prevalent in the Appalachian region mainly Eastern Kentucky and rural Tennessee with a prevalence of 4% and 2.5–3% respectively [1]. Males, people of white race, residents of chronic care institutions [9], and people working with soil (such as persons employed in coal mines and farms) are at the greatest risk of acquiring this disease [10]. A strong association is noted between strongyloidiasis and concurrent immunosuppressive disorders such as HTLV-1 (Human T-cell Lymphotropic Virus – 1) or HIV (Human Immunodeficiency Virus) infection and hematological malignancies [11-13].

## Biology of Strongyloidiasis

**Figure 1:**
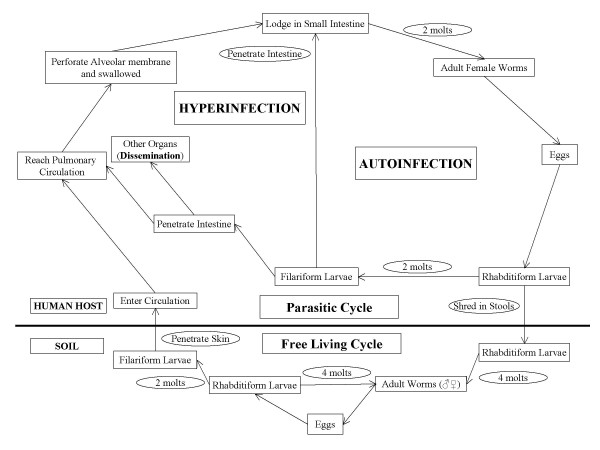
**Life cycle of *Strongyloides stercoralis***. Complete life cycle of *Strongyloides stercoralis *with both parasitic and free living cycles along with the cycle leading to autoinfection and hyperinfection syndrome. The cycle involving the mechanism of autoinfection is shown on the right side of the figure with the cycle elucidating the concept of hyperinfection and disseminated infection on the left side of the figure (adapted from: Division of Parasitic Diseases, National Center for Infectious Diseases, Centers for Disease Control & Prevention; Permission via phone taken from Ms. Melanie at (770) 488–4063).

The life cycle of *Strongyloides stercoralis *is distributed between the free-living and parasitic cycles [10,14]. *Strongyloides stercoralis *is a soil dwelling nematode and may take one of the two cycles depending on the prevalent conditions and turns parasitic in adverse conditions [10]. The free-living cycle begins with the passage of the rhabditiform larvae in the stool, which, on reaching the soil, molt under favorable conditions to become adult free living worms. These worms reproduce sexually and produce rhabditiform larvae in turn, which then molt into filariform larvae. The latter forms infect the human host or develop into free living adult forms and continue the free living cycle as shown in the Figure 1[10,14].

In the parasitic cycle, the rhabditiform larvae once passed in the stool molt only twice and develop into filariform larvae, which are infective to humans. When a susceptible host is available, the infective filariform larvae penetrate the intact skin, travel to the blood stream via subcutaneous lymphatics, and reach the pulmonary circulation. Here they penetrate the alveolar membrane to become air borne, ascend the tracheobronchial tree, are swallowed by the host, reach the small intestine, molt twice more to become adult parthenogenetic females. The females then shed eggs which become rhabditiform larvae and are passed out in the stools, to continue the cycle as shown in the Figure 1[10,14].

Autoinfection is the one of the important characteristic features of the life cycle of *Strongyloides stercoralis*. The various life cycle changes in the case of autoinfection are: the rhabditiform larvae instead of being shed in the stool molt twice in the body of the host (mainly in the intestine) to become filariform larvae that then penetrate the intestinal wall or perianal skin and reach different organs of the body leading to hyperinfection syndrome, if limited to respiratory and gastrointestinal tracts or disseminated infection with involvement of other organ systems.

## Clinical manifestations of Strongyloidiasis

Strongyloidiasis can manifest in a wide spectrum of clinical features ranging from asymptomatic disease, disease with mild initial symptoms, disease with chronic symptoms and acute exacerbation with hyperinfection or dissemination of larvae involving respiratory and gastrointestinal systems or multiple organ systems respectively. Though fatal hyperinfection or dissemination can occur, asymptomatic strongyloidiasis is the most common form of the disease [1,10]. The various clinical manifestations are shown in the Table 2.

**Table 2:** Clinical Manifestations of Strongyloidiasis by Organ System

Organ System	Symptoms	Signs	Investigation	References
Skin	Pruritis, Eruption	Urticaria, Angioedema, Larva Currens, Eruption	CBC with differential counts	
GI	Abdominal pain, Diarrhea, Nausea, Vomiting	Weight loss, Malabsorption, Epigastric tenderness	Stools for parasites, Strongyloid antibody titer	[1]
Pulmonary	Wheezing, Cough, Hemoptysis, Shortness of breath	Wheeze, Rales	Chest X ray, Sputum culture, Sputum for parasites	
CNS	Headache, Altered mental state, Focal seizures, Coma	Meningeal signs, Disorientation	Lumbar puncture and cultures	[1]
Immune/Allergic	Urticaria, Anaphylaxis	Urticarial rash, Larva Currens rash	CBC with differential counts	[65]
Hematological	Fever, Chills, Rigors	Tachycardia, Bacteremia, Septicemia, Eosinophilia	Blood cultures, CBC with differential counts	[1]
Other (Rare)	Peritonitis, Endocarditis, Eosinophilic pleural effusion, Eosinophilic granulomatous enterocolitis			[1] [39] [40]

GI, Gastrointestinal; CNS, Central Nervous System; CBC, Complete Blood Count;

### 1. Initial (acute) manifestations

The initial symptoms happen soon after the entry of the infective filariform larvae into the human host from its extraintestinal migration in the host. Though the acute initial manifestations are not well described [15], the following symptoms are noted in some human infections: serpiginous urticarial rash at the site of entry of the filariform larvae mostly in the legs [1,10,15], cough and tracheal irritation mimicking bronchitis from migration of the larvae through the lungs [10], abdominal cramping with bloating, watery diarrhea and sometimes constipation due to lodging of the larvae and maturation into adult females in the small intestine of the host [10,16-18]. In fact, the most common complaint noted was abdominal bloating [17,18]. As these initial manifestations are vague and mimic multiple other diseases, they are often misdiagnosed and treated symptomatically with the host still harboring the parasite leading to a chronic state of the disease.

### 2. Chronic manifestations

Even though the chronic form of strongyloidiasis is asymptomatic in most cases, mild symptoms involving pulmonary and gastrointestinal systems can happen [10,19]. The various chronic manifestations include nausea, vomiting, epigastric pain with tenderness, intermittent vomiting, diarrhea, constipation, weight loss, asthma-like symptoms, urticaria and distinctive larva currens rash from the subcutaneous migration of larvae [1,10,20-26]. During the asymptomatic stage, the only clinical finding could be eosinophilia [1,10]. Unless physicians have high index of suspicion based on various factors like residence in endemic areas or World War II veterans, there is a high likelihood of misdiagnosis. This requires physicians in the endemic areas to be more aggressive in their investigational workup for eosinophilia. This idea is exemplified by the patient described earlier.

### 3. Hyperinfection syndrome

Immunosuppression, either iatrogenic (for example, use of systemic corticosteroids for chronic obstructive pulmonary disease or asthma, systemic lupus erythematosus, rheumatoid arthritis, autoimmune hemolytic anemia, chronic active hepatitis) [27], or due to intercurrent illness such as HTLV-1 and HIV infection, organ transplantation, and other infectious diseases like kala-azar [28] can increase the risk of hyperinfection syndrome in patients with strongyloidiasis [1,4,21,29-36]. Hyperinfection syndrome is estimated to happen in 1.5 to 2.5% of the patients with strongyloidiasis [37].

Hyperinfection syndrome is not exactly defined, but the hallmark is an increase in the number of larvae in the stool and/or sputum along with manifestations confined to respiratory and gastrointestinal systems along with peritoneum [10]. The hyperinfection syndrome happens from the enormous multiplication and migration of infective larvae especially in an immunosuppressed state. The manifestations of hyperinfection syndrome are divided, based on the system of origin, into intestinal and extraintestinal disease mainly involving the respiratory tract.

The intestinal manifestations include severe cramping abdominal pain, watery diarrhea, weight loss, nausea, vomiting and occasionally gastrointestinal bleeding [10]. Subacute intestinal obstruction can also be caused by strongyloidiasis [38]. The extraintestinal manifestations include mainly asthma-like symptoms such as cough and wheezing, and others such as pneumonia and pulmonary hemorrhage with diffuse bilateral infiltrates on the chest x ray [1,10]. Rare conditions like eosinophilic pleural effusions [39] and eosinophilic granulomatous enterocolitis [40] have also been reported in strongyloidiasis.

### 4. Other manifestations (including disseminated infection)

Even though, most cases of strongyloidiasis are asymptomatic or present with mild symptoms, fatal disseminated infection with involvement of multiple organ systems other than the respiratory and gastrointestinal systems as in hyperinfection syndrome could also occur especially in patients with immunosuppression from systemic steroids [1,4]. Chronic infection and malnutrition also predispose to systemic strongyloidiasis [19]. The mortality from disseminated infection could be up to 87% [1,41,42]. The high mortality rate associated with hyperinfection syndrome and disseminated disease is frequently due to secondary bacterial infections [43,44]. The disseminated infection occurs when the larval load increases, leading to involvement of multiple organs thereby leading to various manifestations along with severe respiratory and gastrointestinal features as mentioned above [19].

The cutaneous manifestations that could occur from dissemination include widespread petechiae and purpura [22,45]. Occasionally this may also present as a pruritic, erythematous, morbiliform eruption [15], or as an intensely itching prurigo [23]. One of the most important and potentially fatal complications that can occur is gram negative bacteremia mainly from pathogens such as *Streptococcus bovis *[43], *Escherichia coli*, *Streptococcus fecalis*, *Klebsiella pneumoniae *[1], or Enterobacter sp. [46] as they become blood borne when the larvae penetrate the intestine [31,43]. Involvement of the central nervous system may lead to headache, altered mental state, seizures and rarely coma [1]. Gram negative bacterial meningitis has also been frequently reported, especially in association immunosuppression [43,47-51].

## Corticosteroids and Strongyloidiasis

**Figure 2:**
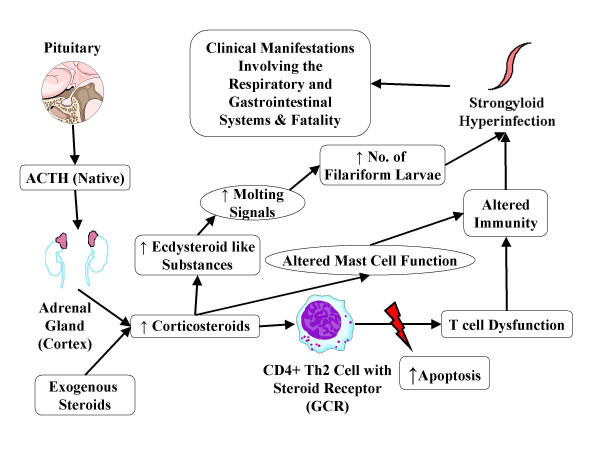
**Corticosteroids and strongyloid hyperinfection syndrome**. The pathophysiological pathway showing the mechanism of corticosteroids leading to strongyloid hyperinfection syndrome and disseminated infection. Corticosteroids along with cortisol act on specific receptors called glucocorticoid receptors (GCRs) available on CD4+ Th_2 _cell membrane causing apoptosis and thus T cell dysfunction. Corticosteroids also increase ecdysteroid like substances in the body which act as molting signals for eggs and rhabditiform larvae, leading to increased number of filariform larvae [41,57].

Multiple case reports indicate a potential increase in the frequency of fatal hyperinfection or disseminated infection with corticosteroid therapy in patients with asymptomatic or mild strongyloidiasis [7,52-56]. Corticosteroids, endogenous as well as exogenous, have been shown to affect the immunity by increasing the apoptosis of Th2 cells, reducing the eosinophil count and inhibiting the mast cell response there by leading to hyperinfection or disseminated infection [1]. It is also proposed that both exogenous and endogenous corticosteroids increase ecdysteroid like substances (naturally occurring sterols with non-hormonal anabolic effects) in the body mainly in the intestinal wall. These substances act as molting signals and lead to increased production of autoinfective filariform larvae leading to hyperinfection and disseminated infection as shown in the Figure 2[41,57]. Siddiqui et al., have demonstrated the presence of steroid receptor on *Strongyloides stercoralis*, which could also play a role in the pathogenesis of hyperinfection syndrome and more systemic disseminated infection associated with corticosteroids, but this area needs further study [41].

## Diagnostic aspects

The important conditions which can confuse the physicians in the diagnosis of strongyloidiasis include other nematode infections. Strongyloidiasis, especially the hyperinfection syndrome and more systemic disseminated infection, can sometimes mimic pneumonia [58], polyarteritis nodosa [59], malignant mediastinal neoplasia [60], eosinophilic folliculitis [61], relapse of lymphoma [62], primary intestinal lymphoma [38], flare up of systemic lupus erythematosus [63], peptic ulcer disease [64], and ulcerative colitis or crohn's disease [40,64].

Due to the asymptomatic nature of intestinal strongyloidiasis, and the risk for hyperinfection, screening of the population in endemic areas especially before considering immunosuppressive therapy is important. There is no one ideal screening or diagnostic test, making strongyloidiasis a difficult infection to detect in humans [18,65].

The most important laboratory finding seen in patients with strongyloidiasis is eosinophilia [65]. Eosinophilia is shown to be 93.5% sensitive with a specificity of 93.1% in high risk populations [2]. But it is also shown that the eosinophil count if used alone is not sufficiently sensitive to screen for strongyloidiasis [18,66] especially in patients with chronic infection who can have low or normal eosinophil counts [65], in people returning from developing countries [66], and in some cases of hyperinfection syndrome and disseminated strongyloidiasis. However, increased peripheral eosinophilia in the case of hyperinfection syndrome could be considered as a good prognostic factor [10]. Eosinophilia is not a cost effective strategy compared to the stool examinations with agar plate culture method and serological testing in detecting strongyloides infection in humans [67].

Diagnosis of strongyloidiasis could be done by serological methods especially in asymptomatic patients with eosinophilia (as in the patient described in this report) or mildly symptomatic patients. These serological tests are also shown to be useful in the diagnosis of strongyloidiasis even in immunocompromised individuals [68]. The serological methods determine the presence of strongyloid antibody in the serum of the human hosts. The antibody could be determined by the following methods: 1) Enzyme-Linked Immuno-Sorbent Assay (ELISA) [69,70], 2) Gelatin Particle Indirect Agglutination (GPIA) [69], and 3) Western Blot Analysis (WBA) [8].

Huaman et al., in their study showed that both ELISA and GPIA are useful in the diagnosis of strongyloidiasis with sensitivities of 74.1% and 98.2%, respectively and specificity of 100% for both studies. As low titers of strongyloid-specific antibodies are noted in hyperinfection along with a low or normal eosinophil count, GPIA is more sensitive than ELISA in detecting the specific immunoglobulin in cases of chronic infection and hyperinfection [69]. New strongyloides-specific antigens are being discovered that may help in the immunodiagnosis of strongyloidiasis in a more sensitive and effective way [71].

The diagnosis of strongyloidiasis can also be made very reliably by observing strongyloid larvae in stool or sputum specimens. But these tests are not accurate as there is fluctuation in the rate of larval excretion especially in stools [8] decreasing the efficacy and accuracy of these tests [72]. Repeated multiple stool specimens should be analyzed to increase the efficacy of the test in the presence of strong suspicion of strongyloidiasis [2,8,73]. Sudharshi et al., in their study have noted stool examinations by formalin-ether concentration method for larvae to be less sensitive in detecting the disease when used alone, especially in non-endemic regions [18].

Microscopic examination of stool specimens is done for strongyloid larvae. This can be done in the following different ways: 1) Simple direct smear with a sensitivity of 0–52% [8], 2) Formalin-ether concentration method with a sensitivity of 13–55% [8], 3) Harada-Mori filter paper culture method with almost equal sensitivity to the above 2 methods [74,75], and 4) Agar plate culture technique with higher sensitivity of 78–100% [8]. Of all the mentioned methods, multiple studies have shown detection of strongyloides larvae in stool specimens is more effective and accurate with agar plate culture technique [4,73-75]. Biopsy with duodenal intubation and Enterotest™ are rarely used, though they have high sensitivity, because they are very cumbersome [76].

## Pathophysiological mechanisms and host defense pathways regulating Strongyloidiasis

Experiments on mice and other animal models have yielded information about the immune mechanisms against nematode parasites and they have also shown that mechanisms differ depending on the host species and the parasite strain used [77]. These experiments support the theory that both T and B cell mediated immunity are required in the immune response to *Strongyloides stercoralis *[78-80].

Increased serum IgE (Immunoglobulin E) levels, eosinophilia in the peripheral blood, increased numbers of mast cells in the intestine, and hyperplasia of goblet cells of the intestine are the responses noted in the nematode infections of the gastrointestinal tract [81-83]. Eosinophils, intestinal mast cells and goblet cells, IgE, type 2 helper T (Th_2_) cells and cytokines such as IL-4 (Interleukin-4), IL-5 (Interleukin-5), IL-6 (Interleukin-6), IL-9 (Interleukin-9), IL-10 (Interleukin-10), and IL-13 (Interleukin-13) are shown to be the mediators of immunity against *Strongyloides stercoralis *[1,83-91]. The various types of defense mechanisms against strongyloides larvae are shown in Table 3.

**Table 3:** Various Types of Immunity in Strongyloidiasis

**Type of Immunity**	**Mediators**	**Reference**
T Cell Mediated Immunity	CD4+ Cells	[11,12,92]
Th_2 _Cellular Immunity	IL-4, IL-5	[77,81]
Humoral Immunity	IgM, IgG, IgA, IgE	[95,98]
Antibody Dependent Cellular Cytotoxicity	IgM, IgG, Eosinophils, Neutrophils	[91,98,99]
Mucosal Immunity	Mast Cells, Goblet Cells	[81,82,87]
Complement System	Complement activation	[99]

CD, Cluster Differentiation; Th2, Type 2 T Helper; IL, Interleukin; IgM, Immunoglobulin M; IgG, Immunoglobulin G; IgA, Immunoglobulin A; IgE, Immunoglobulin E;

There are 2 broad types of defense mechanisms in the case of *S. stercoralis *– mechanisms against the filariform larvae, which infect the human host for the first time and mechanisms against the host adopted larvae that cause autoinfection. The following defense mechanisms may function in one or both of the above types.

T cell mediated immunity, especially involving CD4+ cells, has been shown to be important in the defense against *Strongyloides stercoralis *larvae using a rodent model [92]. In humans, as the infection is more prevalent in hematological malignancies and HIV infected people, it is proposed that T cell immunity involving CD4+ cells plays a role in the defense against strongyloidiasis [11,12]. In a study reported by Trajman et al., the detection of T cell-dependent serum-specific IgA and IgE antibodies indicates the importance of T cell immunity in *S. stercoralis *infection [84]. It has been shown that contact with immune cells is required for immunity against strongyloides larvae (as reported in a rodent model using the human parasite, *Strongyloides stercoralis*) [93].

Type 2 T helper (Th_2_) cells that stimulate eosinophils, IgE production, mast cells, and goblet cells by producing IL-4, IL-5, IL-9 [91], IL-10 and IL-13, are important in the defense against strongyloides infection [1,81,84,91,92]. This has been shown, for example, in people with co-infection with HTLV-1 which decreases Th_2 _cell-mediated immunity, thereby increasing the risk for hyperinfection syndrome or disseminated infection [94].

In severe strongyloidiasis, no significant change in the T cell numbers is noted compared with asymptomatic or mildly symptomatic disease [95] indicating the importance of other immune mechanisms. It is also reported that infected patients elicit a decreased lymphocyte blastogenic response to larval *Strongyloides stercoralis *antigens suggesting presence of factors in the serum of patients that are inhibitory to the function of cell mediated immunity [84,86].

Controversy exists over whether one cytokine is more important than another in the resistance against nematodes even in animal studies. IL-3 (Interleukin-3) has been shown in multiple previous studies as a potent mast cell synthesis stimulator. But, Kobayashi et al., using a rodent model showed *Strongyloides venezuelensis *can stimulate mastocytosis without IL-3 indicating a role of other mechanisms [90]. IL-4 is an important regulator of IgE antibody production and mast cell activation [88]. Urban et al., using an animal model with *Heligmosomoides polygyrus *have shown that IL-4 is an important factor in the defense against gastrointestinal nematode infections [88]. Watanabe et al., showed the importance of IL-4 in the induction and maintenance of intestinal mast cells with *Strongyloides ratti *in mice [81]. As no human studies are available indicating the importance of IL-4, and with the exact role of IL-4 in helminth infection being unknown, it would be premature to consider IL-4 as an important factor in the defense against strongyloidiasis [91]. IL-5 is important in differentiation, maturation and survival of eosinophils [77] by regulating the eosinophil precursors in the bone marrow [88]. Herbert et al., showed the possible roles played by IL-5 in innate as well as adaptive immunity against human parasite larvae in mice, by inducing eosinophil production during innate immunity and IgM (Immunoglobulin M) production in the adaptive immune response. Porto et al., in their effort to demonstrate the effect of HTLV-1 in patients with strongyloidiasis, noticed increased IL-5 in patients with strongyloidiasis [96]. These studies involving the human parasite-rodent models and human parasite-humans indicate a very important part played by IL-5 in the immunity against strongyloides infection especially by inducing eosinophil differentiation, maturation and survival.

Humoral immunity mainly includes the defense mechanisms with the production of immunoglobulins by plasma cells. Herbert et al., in their experiment with mice using human parasites, showed that B cells play no part in the defense against primary infection but play an important role in subsequent challenge infections with a resultant increase in parasite specific immunoglobulin, mainly IgM [78].

Immunoglobulins are shown to play an important role in the defense against strongyloidiasis. Even though strongyloid-specific antibodies are noted in the infection, they do not provide the required immunity without the help of other defense mechanisms. There is increasing evidence that IgE mediated activation of accessory cells can play an important role in the resistance against parasitic infection [82]. Even though IgE is increased in the helminth infections, most of that is non-specific and may block the development of the host defense mechanism by saturating IgE receptors on effector cells [91]. Brigandi and coworkers while experimenting with mice and *Strongyloides stercoralis *noticed IgE along with IgA to have no effect on killing of infective larvae. In humans, IgE antibodies are specifically formed against filariform larvae [97]. However, Badaro et al., showed that no correlation exists between the total IgE, specific IgE and IgG (Immunoglobulin G) antibody quantities and the clinical severity of strongyloidiasis. As humans differ from rodents and experimental models, IgE-mediated anti-parasite immunity is controversial [91]. As there is an increase in IgE levels in strongyloides infection both in human subjects with the infection as well as in experimental models, it is likely that IgE plays a pivotal role, but the exact nature of this role is currently unclear. However, determination of IgE levels with radioallergosorbant test (RAST) could be helpful in the immunological evaluation of the patient [97].

As it is shown in animal models, IgG and IgM can passively transfer immunity against human parasite larvae in the presence of a well functioning complement system and neutrophils [77,93,98,99]. In severe strongyloidiasis, IgA (Immunoglobulin A), IgG and IgM levels were significantly lower compared with asymptomatic or mildly symptomatic strongyloidiasis [95]. It is also shown, in mouse model with the human parasite, host adopted or autoinfective filariform larvae have different surface antigens and IgM specific for primary infective filariform larvae is not effective against autoinfective larvae [100]. Lack of host response to autoinfective larvae along with lack of specific IgM antibodies could play a role in autoinfection and thus in hyperinfection syndrome and disseminated infection.

Antibody Dependent Cellular Cytotoxicity (ADCC) is proposed as one of the defense mechanisms against the nematodes especially in rodent experiments involving human parasite larvae with IgE activation of eosinophils [1,91] as well as IgG activated ADCC involving neutrophils [98]. As shown in the experiments on mice with *Strongyloides stercoralis *larvae, ADCC may play a role in the immunity against live infective larvae but not against injected larval antigens [98,101]. However, Kerepesi et al., with experiments on mice and human parasite larvae indicated ADCC is not required for larval killing [102]. Insufficient and controversial results do not substantiate the role played by ADCC in the immunity against strongyloides, requiring further research to demonstrate its importance.

Eosinophils are bone marrow derived granulocytes which can secrete highly toxic substances contained in their granules in the wake of infections and allergic reactions [82]. Eosinophil production is activated by IL-5 released by the Th_2 _cells and in turn eosinophils also release lymphocyte active cytokines that can stimulate and affect the functioning of lymphocytes [82,103]. In experimental studies using mice and human parasite larvae, an increase in eosinophils is seen with parasite killing in nematode infections [93,104]. Eosinophils seem to be directly involved in the destruction of helminths and their migrating larvae as they penetrate the intestinal mucosa [83,91] as shown in the animal studies, especially in mice in the presence of specific antibodies [82,91]. Though eosinophils can cause destruction of helminth larvae, especially host-adopted filariform larvae [104], it is not sufficient for complete protection [91]. In humans, their role in the defense against *S. stercoralis *is controversial.

Even though eosinophils are noted to be important in defense against *Strongyloides stercoralis*, significantly lower eosinophil counts are noted in severe strongyloidiasis [95] indicating a possibility of suppression of eosinophils especially in disseminated infections. Neutrophils are important in immunity against infective larvae of *Strongyloides stercoralis *in mice [98,99] which could also play a role in human strongyloidiasis. They are shown to have a controversial effect through the ADCC mechanism with the help of IgG as mentioned earlier.

All nematode infections elicit the same intestinal phenomena with different effector mechanisms [81,91]. Strongyloides infections are difficult to eradicate completely mainly because of autoinfection. As this nematode resides in the small intestine [1,10] of the human beings, local mucosal immunity along with inflammatory changes may play a role in their eradication [87]. Many studies are done on mice to evaluate the mucosal responses to gastrointestinal nematode infections. There are different mechanisms in which the local mucosal immunity defends against the infection manifesting in different forms like expulsion of the adult worms, decrease in the length of the worms, reduction in fecundity of the female worms, and failure of infective larvae to establish [87]. The above said mucosal defense mechanisms are mediated by both humoral and cell mediated immunity.

Studies with animal models and *Strongyloides ratti *showed the important role played by goblet cells in the expulsion of the parasite from the GI tract [81]. These goblet cells were induced by IL-13 secreted by Th_2 _cells [81]. Intestinal mastocytosis, induced by IL-3 [90], is observed in many nematode infections. Mast cells are also shown to play an important role in the defense against parasite infestations [82] and are considered as the effector cells against Strongyloides species even in humans [90]. Experiments on mice with Strongyloides species have shown the importance of intestinal mastocytosis in the expulsion of the worm from the gastrointestinal tract.

Trajman et al., in their study, have noted that intestinal response in the form of expulsion is seen in rodent experiments and could not be confirmed in human beings. No change in the jejunal morphology, different T cell subset numbers, mast cells, eosinophils and goblet cells were noted along with lack of cell activation in the mucosa as shown by the absence of CD25+ cells. However, they noted a decrease in the number of mature macrophages and dividing enterocytes in the crypts of the intestinal wall [84]. Absence of immune response might be the reason for preservation of architecture of the mucosa and absence of immune mediated diarrhea in *Strongyloides stercoralis *infection. This indicates the controversial role of the mucosal immunity played against strongyloides infections necessitating further studies especially involving human subjects.

Activation of both classical and alternate pathway of the complement system [102,105] with chemoattraction and binding of the granulocytes to the infective larvae has also been shown to play a role in the immunity against *S. stercoralis *in mice [99,102]. Machado et al., with their experiment on mice have indicated the potential role played by the leukotrienes as immunostimulants against strongyloides [106]. But the specific role of complement and leukotrienes in strongyloides infections is not proven even in experimental animals.

## Management

Albendazole, mebendazole, thiabendazole and ivermectin have shown to be effective on *Strongyloides stercoralis *[107-109]. Recently, there has been a change in the treatment of strongyloidiasis with more studies showing ivermectin as the drug of choice. In a randomized control trial done by Gann et al., comparing ivermectin with thiabendazole, the investigators showed that one dose of ivermectin is as effective as prolonged thiabendazole in the treatment of strongyloidiasis with less adverse side effects [107]. Ivermectin is shown to be very effective in the treatment of strongyloidiasis [109] as well as in the treatment of hyperinfection syndrome with predisposing conditions like AIDS (Acquired Immunodeficiency Syndrome) [33]. Treatment of strongyloidiasis has been shown to improve the cutaneous manifestations [25] as well as asthma-like symptoms associated with the nematode [110].

In *Strongyloides stercoralis*-infected patients presenting with asthma-like symptoms, physicians should be cautious in using leukotriene synthesis inhibitors. The use of leukotriene synthesis inhibitors may worsen the infection as leukotrienes are shown to play a potential role in the immunity against strongyloides infection in murine animal models [106] and possibly in human subjects. More research is needed in this aspect before limiting the use of leukotriene synthesis inhibitors.

Relapses, especially involving gastrointestinal tract, were noted even after treatment of strongyloidiasis. For the chronicity of the infection and possibility of occurrence of dangerous exacerbations, diagnostic surveillance is recommended to prevent the occurrence of fatal hyperinfection [65]. The surveillance could be done in the form of repeat eosinophil counts along with serological surveillance with serial antibody titers and multiple stool specimens for larvae.

The efficacy of the treatment depends on many factors like immunodeficiency, co-infection with HTLV-1, use of corticosteroids and presence of bowel ileus that can decrease the efficacy of the drugs used in the treatment of strongyloidiasis [19,94]. Monitoring the response to treatment could be very difficult with detection of strongyloid larvae in the stool specimen because of the inconsistent shedding of the larvae [72].

Loutfy et al., in their study have suggested that serological tests mainly EIA with serial eosinophil counts could be used to monitor the response to the treatment [76]. In a study involving 3 patients with strongyloidiasis, serial total IgE levels decreased after effective treatment indicating a possibility of using serial total IgE levels in monitoring the response to treatment [111]. As no prospective studies were done in this direction, it would be very difficult to determine which test would be the test-of-cure for strongyloidiasis [76]. Failure to respond to treatment or recurrence of hyperinfection syndrome is an indication to look for latent or asymptomatic HTLV-1 infection [112, 113].

Personal hygienic measures like proper protection of skin to prevent contact with infected soil, community level hygienic measures like proper disposal of human excreta, community education about protective and hygienic measures, and prompt treatment of diagnosed cases would help in the prevention of the disease [44].

Vaccines, which are effective against infective stage filariform larvae could be a possibility based on the experimental evidence in mice, especially if composed of multiple antigens [98,99,102,104]. Further studies mainly involving voluntary human subjects are required to confirm this possibility.

## Conclusion

Strongyloidiasis is a nematode infection with a tendency to become chronic with fatal complications of hyperinfection syndrome and disseminated infection along with a host of other potential complications like gram-negative bacteremia and meningitis. As the infection is mostly chronic and asymptomatic, and there is no specific ideal test to diagnose the disease, it still tends to be a diagnostically elusive disease even in the present era.

Non-endemic regions of the world have endemic pockets for strongyloidiasis, like rural Tennessee and Kentucky in the USA, making the disease more important especially in view of potentially fatal hyperinfection syndrome and disseminated infection. As most cases of hyperinfection syndrome and disseminated strongyloidiasis happen in immunocompromised individuals, especially those who are taking systemic steroids, physicians in the endemic areas should be aware of the bizarre manifestations of the disease that can mimic other diseases leading to misdiagnosis and medical errors.

## Competing interests

The author(s) declare that they have no competing interest.

## Authors' contributions

RSV carried the literature review, drafted the manuscript, and prepared the tables and figures. DSC reviewed and edited the manuscript for content and style. KG supplied the manuscript outline, and reviewed the manuscript for final submission.
